# Contagious caprine pleuropneumonia – a comprehensive review

**DOI:** 10.1080/01652176.2019.1580826

**Published:** 2019-04-01

**Authors:** Mohd. Iqbal Yatoo, Oveas Raffiq Parray, Shah Tauseef Bashir, Muheet, Riyaz Ahmed Bhat, Arumugam Gopalakrishnan, Kumaragurubaran Karthik, Kuldeep Dhama, Shoor Vir Singh

**Affiliations:** a Mycoplasma Laboratory, Division of Veterinary Clinical Complex Faculty of Veterinary Sciences and Animal Husbandry, Shuhama, Srinagar, India;; b Department of Molecular and Integrative Physiology, University of Illinois, Urbana-Champaign, IL, USA;; c Department of Veterinary Clinical Medicine, Madras Veterinary College Tamil Nadu Veterinary and Animal Sciences University, Chennai, India;; d Central University Laboratory, Tamil Nadu Veterinary and Animal Sciences University, Chennai, India;; e Division of Pathology, ICAR-Indian Veterinary Research Institute, Izatnagar, Bareilly, India;; f Animal Health Division, Central Institute for Research on Goats (CIRG), Mathura, India

**Keywords:** Goat, caprine, pleuropneumonia, Mycoplasma, vaccine

## Abstract

Contagious caprine pleuropneumonia (CCPP) is a serious disease of goats, occasionally sheep and wild ruminants, caused by *Mycoplasma capricolum* subspecies *capripneumoniae* (Mccp). The disease is characterized by severe serofibrinous pleuropneumonia, very high morbidity (∼100%), and mortality (80–100%). CCPP affects goats in more than 40 countries of the world thereby posing a serious threat to goat farming around the globe. The characteristic clinical signs of CCPP are severe respiratory distress associated with sero-mucoid nasal discharge, coughing, dyspnea, pyrexia, pleurodynia, and general malaise. In later stages, severe lobar fibrinous pleuropneumonia, profuse fluid accumulation in pleural cavity, severe congestion of lungs and adhesion formation is observed. Mycoplasmal antigen interactions with host immune system and its role in CCPP pathogenesis are not clearly understood. CCPP is not a zoonotic disease. Diagnosis has overcome cumbersome and lengthy conventional tests involving culture, isolation, and identification by advanced serological (LAT, cELISA) or gene-based amplification of DNA (PCR, RFLP, and hybridization) and sequencing. The latex agglutination test (LAT) is rapid, simple, and better test for field and real-time diagnosis applicable to whole blood or serum and is more sensitive than the CFT and easier than the cELISA. Moreover, the studies on antibiotic sensitivity and exploration of novel antibiotics (fluoroquinolones, macrolides) can help in better therapeutic management besides preventing menace of antibiotic resistance. Re-visiting conventional prophylactic measures focussing on developing novel strain-based or recombinant vaccines using specific antigens (capsular or cellular) should be the most important strategy for controlling the disease worldwide.

## 1. Introduction

Contagious caprine pleuropneumonia (CCPP) is a highly contagious and rapidly spreading mycoplasmal disease that affects a vast majority of goat populations. Considering the importance of this disease, worldwide measures are being taken for understanding this disease for better diagnosis, prevention, and control. Periodically, the disease dynamics are reviewed by researchers and agencies of national and international mandate and repute for keeping vigil on this transboundary emerging threat that endangers naive goat populations in various disease-free countries from the surrounding infected herds of neighboring and mutually cooperative countries. The chronological reviews on CCPP by Rurangirwa et al. (1987), Thiaucourt et al. (1992), Thiaucourt et al. (1996), Thiaucourt and Bolske (1996), Kusiluka (2002), Nicholas and Churchward (2012), Samiullah (2013), AU-IBAR (2013), Prats-van der Ham et al. (2015), Asmare et al. (2016), OIE (2017), and EFSA AHAW Panel et al. (2017) suggest the changing trend in need of future strategies with initial reviews focusing on general perspectives of the disease including status of disease, diagnosis, treatment, and prevention and current reviews focusing on novel diagnostic and prophylactic measures against this emerging disease for global prevention and control besides minimizing risk of antimicrobial resistance and eradicating carrier state.

Our effort in this regard has been to revive the study of the disease dynamics including epidemiology, pathophysiology, current status, and future prospects with emphasis on diagnostics, therapeutics, and prophylactics and future challenges or threats.

### 1.1. Economic losses

CCPP is considered as one of the most severe and highly infectious disease of goats. It results in heavy economic losses to countries involved in goat farming especially in Africa, Asia and the Middle East (Jones and Wood 1988; Wesonga et al. 2004; OIE 2014). The disease is one among the mycoplasmal infections resulting in significant losses in almost 40 countries, and morbidity and mortality can be as high as 100% especially in exotic breeds (DaMassa et al. 1992; OIE 2014). In naive and native herds, 100% morbidity and 80% mortality have been noted. It is estimated that the total yearly cost of CCPP is about US$507 million in endemic areas thus involving major economic losses. Economic losses are both by morbidity, mortality and decline or loss of production performance in addition to costs involved in prevention, control and treatment (Yatoo et al. 2018; Parray et al. 2019). Morbidity causes constraints in livestock management, overburdens with costs of treatment, and imposes restriction on trade or transport. Mortality causes direct loss by death of the valuable animals. Diseased animals are usually culled in developed countries, which is not possible in under developed and developing countries like India (Yatoo et al. 2018; Parray et al. 2019). Loss on production performance is severe. As an illustration, the disease causes huge economic losses to Changpas, native inhabitants of Changthang, Ladakh, India who are traditional Pashmina farmers and are directly dependent (90–100%) on the traditional and nationally important venture-Pashmina farming (Yatoo et al. 2014, 2018) and contribute 80% (45–50 tons) to national output (60 tons) of Pashmina. A loss of around 30% in Pashmina yield in CCPP affected goats and a benefit cost ratio of 0.79 in untreated animals against 8.76 in treated animals has been reported (Yatoo et al. 2018).

### 1.2. Threat to other countries

CCPP is emerging as a serious threat to other countries which either never encountered this highly contagious disease or are at risk of contracting the disease because of regular trade with the affected countries or the adjacent neighborhood by geographical location (Manso-Silván et al. 2011; OIE 2017). As CCPP is noted in Turkey, which borders Greece and Bulgaria of the European Union, and Armenia and Georgia on the Russian side since 2003 (Ozdemir et al. 2005; Cetinkaya et al. 2009), there is a constant risk of spread of CCPP to Europe and Russia (Manso-Silván et al. 2011; EFSA AHAW Panel et al. 2017). Mycoplasma outbreaks in Greece have been suspected but not confirmed (OIE 2008). The occurrence of CCPP in Africa (AU-IBAR 2011), Middle East (OIE 2008; OIE 2009), and Asia (Walker 1914; Li et al. 2007; Awan et al. 2010; Srivastava et al. 2010; Parray et al. 2019) in both domestic and wild animals (Li et al. 2007; Wang et al. 2011; Chu et al. 2011; Yu et al. 2013; Huang et al. 2013) has been the usual phenomenon, but spread to other areas and continents has provoked serious note for disease thus enforcing OIE to place it under List B diseases and a notifiable one in these regions. Recently, CCPP has been noted in China and Tajikistan (Amirbekov et al. 2010; Manso-Silván et al. 2011; Wang et al. 2014). In Europe, South, Central and North America and Oceania, this disease has not been reported or information is not available (OIE 2009; EFSA AHAW Panel et al. 2017). However, recently some reports of CCPP in few countries have surfaced (Prats-van der Ham et al. 2015; Dudek et al. 2016). Nicholas et al. (2008) previously opinioned about the chances of spread. Hence, CCPP has been identified as an emerging infectious disease recently and poses transboundary epidemic threat (Nicholas and Churchward 2012; Prats-van der Ham et al. 2015; EFSA AHAW Panel et al. 2017). Adequate measures need to be adopted for early prevention and control of the disease both in endemic or infected countries and the countries at risk.

### 1.3. Historical perspectives and current risks and outbreaks of contagious caprine pleuropneumonia

CCPP, also called as ‘bou frida’ in Algeria, was first described by Thomas in 1873 and indicating involvement of one lung in the disease (Thomas 1873; Castelet 1906). Being endemic in most of the places, initially it was not considered as a contagious disease rather climatic conditions were considered for disease occurrence. The major outbreak of CCPP in South Africa in 1881 was the main reason of exploring contagiousness of the disease by Duncan Hutcheon, colonial veterinary surgeon, as infection was transported with goats from Turkey (Hutcheon 1881; Hutcheon 1889). Lefèvre et al. (1987a) isolated Mycoplasma species F38 isolated in Chad. In 1906, Castelet (1906) noted CCPP in Algeria and in 1914, a report of occurrence of CCPP in India also appeared (Walker 1914). The etiology of CCPP remained unclear for quite some time. Around 1976, the etiology of CCPP was investigated and Mycoplasma F38 was isolated for the first time *in vitro* (MacOwan and Minette 1976; McMartin et al. 1980) and was later officially named *Mycoplasma capricolum* subspecies *capripneumoniae* in 1993 (Leach et al. 1993). In between, analyzing the large-scale devastating effect of CCPP on goat farming in various countries, researchers focused on CCPP studies in various parts of the world. In Greece, a CCPP outbreak was noted from 1920 to 1930 (Stylianopoulos 1933) with morbidity of 98%, which was lately controlled and eradicated. This occurrence of CCPP was suspected due to the transport of goats from Turkey. However, the exact cause of the disease remained unknown (Nicholas and Churchward 2012). CCPP in the form of acute and chronic disease was reported in Kenya by MacOwan and Minette (1976) and MacOwan (1976), respectively. Analysing the importance of mycoplasmal diseases in goats, Gee (1977) reviewed caprine mycoplasmosis. Of importance, CCPP was reviewed by McMartin et al. (1980). Earlier, an outbreak of CCPP was reported in goats in Gumel, Nigeria (Okoh and Kaldas 1980).

Other notable historical perspectives associated with CCPP are the insights on the spreading of disease to disease-free areas from time to time and frequent outbreaks in many countries where the disease was not present previously. This is particularly of importance in the face of the emerging threat of spread to the European Union as CCPP has been noted in the Thrace region (Ozdemir et al. 2005, 2018) and eastern region of Turkey (Cetinkaya et al. 2009), which borders with states of Europe on one side and Russia on the other side. Hence, CCPP may probably be present in Armenia and Georgia also, being neighbor to Russia which may extend to southern Russia (EFSA AHAW Panel et al. 2017). A large number of outbreaks in countries where it was not so much prevalent previously and a rise in the number of outbreaks in countries that historically have had the disease, also suggest rise in incidence. It is recorded that there were nearly 478 outbreaks from 2006 to 2007 in Iran, affecting 16,000 goats, 38 outbreaks in Ethiopia, 600 outbreaks from 2008 to 2009 in Oman, affecting 30,000 goats, which is an alarming example (OIE 2008, 2009). In 2009 alone, the number of CCPP outbreaks in Tajikistan (4 outbreaks, affecting 166 goats), Tanzania (10 outbreaks affecting 200 goats), Yemen (12 outbreaks affecting 800 goats), and Mauritius (affecting 300 goats) were noted (Srivastava et al. 2010; EFSA AHAW Panel et al. 2017). From 2008 on, frequent outbreaks of CCPP were noted by Awan et al. (2009, 2010, 2012) in Pakistan. Recently, seven countries in Africa reported the occurrence of CCPP. It has started spreading from central and eastern Africa to other parts (AU-IBAR 2011). At present, goat populations in more than 40 countries are affected with CCPP and sporadic outbreaks or cases of CCPP are reported from many more countries from time to time. Hence, CCPP is becoming a novel emerging and rapidly spreading disease in most parts of the world. However, difficulties and financial bindings in culture, isolation and other improved identification systems limits proper reporting and the actual distribution of spread on various continents. For proper reporting, identification of etiological agent is important.

On the American continent, there are no reports of CCPP, but of related species of *Mycoplasma* (Nicholas et al. 2008). Given the difficulties in culture and isolation, frequent observation of field cases of the disease, and need for early rapid and convenient diagnosis, sero-molecular diagnosis has become common from the beginning of the twenty-first century and has added a new perspective of CCPP focusing on diagnosis. In the recent past, seroepidemiological studies were conducted by Hadush et al. (2009) in Northern Ethiopia, Bett et al. (2009) in Kenya and molecular epidemiology in Balochistan, Pakistan by Awan et al. (2010). Overcoming the historical limitations of culture and isolation and with advancement in technology in the current times, various diagnostics are being applied in CCPP diagnosis like DNA sequencing (Manso-Silván et al. 2011), competitive enzyme-linked immunosorbent assay (cELISA) (Peyraud et al. 2014), recombinase polymerase amplification (RPA) assay (Liljander et al. 2015), multiplex polymerase chain reaction (PCR) (Settypalli et al. 2016), and ultrasonography (USG) (Tharwat and Al-Sobayil 2017). Simultaneously, in the current times, new arenas and possibilities and risks associated with CCPP are being explored, like the one of exploring novel vaccine candidates (March et al. 2002; Ping et al. 2014; Thankappan et al. 2017; Thiaucourt et al. 2018), antibiotics and antibiotic resistance, which is already in rise in different mycoplasma (Prats-van der Ham et al. 2017; Tatay-Dualde et al. 2017; Yatoo et al. 2018).

So, considering the historical perspectives of occurrence we provide information about the epidemiology, clinical presentation, pathogenesis, and diagnostic advancements in CCPP.

## 2. Epidemiology

### 2.1. Etiology

CCPP is caused by *Mycoplasma capricolum* subspecies *capripneumoniae* abbreviated as Mccp. Previously, it was known as *Mycoplasma* biotype F38 (Leach et al. 1993). These pathogenic bacteria belong to the class Mollicutes, which lack cell wall but have galactan and small genomes (0.58–1.35 Mb). They have limited biosynthetic capability and cause a number of infections in animals (Nicholas and Baker 1998; Razin et al. 1998). The four lineages of mycoplasma correspond to different geographic regions. Mccp is placed in *Mycoplasma mycoides* cluster and has different species and subspecies, namely *Mycoplasma mycoides* subsp. *mycoides* large colony strains (MmmLC), *Mycoplasma mycoides* subsp. *mycoides* small colony strains (MmmSC), *Mycoplasma* sp. bovine group 7 of Leach (Mbg7), *Mycoplasma capricolum* subsp. *capricolum* (Mcc), and *Mycoplasma mycoides* subsp. *capri* (Mmc) (Cottew et al. 1987; Manso-Silván et al. 2009). Of these members, some cause similar diseases in sheep and goats but have extrapulmonary involvements also. Disease caused by *M. mycoides* subsp. *capri* are different than CCPP as was previously erroneously believed (Cottew et al. 1987; ICSBSTM 1988; Leach et al. 1989).

Different studies have been conducted to reveal the taxonomic relationships between the F38 group of caprine mycoplasmas especially *M. capripneumonia* and *M. capricolum* (Cottew et al. 1987; ICSBSTM 1988; Leach et al. 1989). Close phylogenic relationship among mycoides cluster, especially Mccp, have been reported (Ostrowski et al. 2011). A tritium-labeled DNA-based hybridization study revealed the taxonomic relation between these mycoplasmas (Bonnet et al. 1993), principally because of serological, genomic, and other similar properties between these mycoplasmas (Cottew et al. 1987; ICSBSTM 1988; Leach et al. 1989; Bonnet et al. 1993). Mccp can still be differentiated from other members based on cultural and colony characteristics, classical biochemical, serological and especially molecular tests (Razin et al. 1998; Maigre et al. 2008; Awan et al. 2010). Within Mccp, different strains like strain F38, M1601, 9231-Abomsa, ILRI181 (Chu et al. 2011; Dupuy and Thiaucourt 2014; Falquet et al. 2014; Dupuy et al. 2015) and biochemical groups namely organic acid-oxidizing group and glycerol and the glucose-oxidizing group (Soayfane et al. 2018) have been identified.

### 2.2. Host

Goats (*Capra hircus*) are considered naturally susceptible domestic species, and sheep can also be affected (Bolske et al. 1995), which is yet to be well established. Bovidae family are also considered susceptible (Thiaucourt and Bolske 1996; Shiferaw et al. 2006; Arif et al. 2007; Manso-Silván et al. 2011; Yu et al. 2013; EFSA AHAW Panel et al. 2017). In addition, many wild animal species have been found naturally susceptible to CCPP. Various wild animal families or orders among ungulates are quoted as susceptible too (EFSA AHAW Panel et al. 2017; OIE 2017). CCPP has been reported from various wild animals like wild sheep, wild goat, gazelle, Tibetan antelope, Arabian oryx and sand gazelles (Arif et al. 2007; Yu et al. 2013; Chaber et al. 2014; Lignereux et al. 2018). Though CCPP may affect other wild species, the susceptibility of these species to Mccp has not been worked out (EFSA AHAW Panel et al. 2017).

Sheep may act as the domestic-reservoir species, but this role is not clear. Both wild animals and sheep can get infected from affected goats. Initially, Mccp was isolated in sheep in Kenya (Litamoi et al. 1990), followed by isolation in Uganda (Bolske et al. 1995). In Ethiopia, it was isolated from sheep by Shiferaw et al. (2006). Presently, antibodies against Mccp have also been found in sheep (Mbyuzi et al. 2014). This suggests the multiplication and immunological response in sheep against Mccp even though its long-term persistence is questionable. Our observation of close confinement of infected Pashmina goats with sheep in Changthang, India could not yield an infection in sheep, but antibodies sufficient for agglutination were detected under field conditions (Unpublished data).

Wild reservoir species may include wild ruminants. However, their role as a reservoir or dead-end host for this disease is not clear. Our observation of wild animal (ibex, aragali)-Pashmina goat interaction could not predict any effect on the occurrence of CCPP although improvement in diagnostic aids may help in the detection of CCPP in wildlife (Arif et al. 2007; Yu et al. 2013; Chaber et al. 2014; Lignereux et al. 2018). Further, close contact between affected domestic or wild species can predispose goats to CCPP infection (Arif et al. 2007; Yu et al. 2013; Chaber et al. 2014; Lignereux et al. 2018).

### 2.3. Transmission

Inhalation of infected aerosols is the main route of transmission. The main source of contamination is direct contact with affected animals (Thiaucourt et al. 1996; OIE 2017). Airborne transmission can result in distant spread (Lignereux et al. 2018) with a 50 m distant transmission reported (Lignereux et al. 2018). Infected objects, vectors, fomites and animal products are yet to be known in transmission role (EFSA AHAW Panel et al. 2017). Under cold, moist and overcrowded environment pathogen can persist longer and may lead to severe outbreaks. Shorter survival time (3–14 days) in external environment limits transmission of Mccp (OIE 2009). Higher temperature inactivates Mccp rapidly (within 60 min at 56 °C and within 2 min at 60 °C). However, low temperature prolongs survival. Mccp can survive for 10 years in frozen infected pleural fluid (OIE 2009). Moisture (Justice-Allen 2010) and humidity (Wright et al. 1968) also affect survival and hence transmission of mycoplasma.

### 2.4. Seasonal occurrence

Climatic factors have often been associated to the onset of CCPP outbreaks. In North Africa, the disease appeared more often in the winter (Castelet 1906; Curasson 1942). In Oman, CCPP outbreaks seemed to be more frequent in January, when the lowest temperatures and highest pluviometry are recorded, and in July, when the highest temperatures are registered (Jones and Wood 1988). Also, frequent outbreaks of CCPP in Pashmina goats during winter season in Changthang, Ladakh, India have been observed, especially starting from November–December to March–April where there is an extended winter (October to May) (Yatoo et al. 2018; Parray et al. 2019). Though cold weather is considered as the main predisposing factor, stress of any kind like transport, nutritional or climatic stress can predispose the animals to CCPP (Thomas 1873; Parray et al. 2019).

### 2.5. Persistence in environment and animals and affecting factors

Persistence in environment and animals may be related to conduciveness of the environment, concentration of pathogen, type of breed, herd density or immunity of the animals. The duration of infection is quite variable though recent reports suggest shorter persistence (hours to days) in the environment and longer in affected animals (weeks to months) (Rurangirwa and McGuire 1996; Thiaucourt et al. 1996; March et al. 2000; Wesonga et al. 2004; EFSA AHAW Panel et al. 2017). Mccp being more fragile does not survive outside the animal or in the external environment for long time. Hence, survival in the environment is a less likely event (OIE 2009; Radostitis et al. 2009; EFSA AHAW Panel et al. 2017). On an average, 3 days in tropical areas and 2 weeks in temperate areas is the probable period of Mccp survival in external environment (OIE 2009). Poor survival in environment due to more fragility can be attributed to both pathogen-related factors like lack of cell wall and external factors such as ultraviolet radiation, which can inactivate Mccp quickly (OIE 2009). However, with adaptable changes some environmental survival mechanisms as noted in other mycoplasma species cannot be ruled out in future (McAuliffe et al. 2006).

Actual period of infectiousness in animals post infection is not known. Duration of the infectious period is about 19 days *in silico* model, which may vary in *in vivo* models (Lignereux et al. 2018). As in acute stage with maximum concentration of Mccp in exudate (10^9^ Mccp/ml), persistence of infection may be 5–10 days if the animal survives or until recovery in chronic stages or development of sterile immunity. However, exact data on persistence of infection are not published yet (Spickler 2015; EFSA Panel et al. 2017). In experimentally infected goats, Mccp has been isolated on days 9 and 16 but not on any later occasions (Wesonga et al. 1998). Affected but surviving animals remain infectious until complete recovery occurs. Among the long-term survivors there is no evidence of a carrier state though they may have lesions (Wesonga et al. 1998). Mccp may persist in chronic, latent carriers including sheep and goats that recover from CCPP but without becoming bacteriologically sterile (McMartin et al. 1980; Lefèvre et al. 1987b; Wesonga et al. 1993; Thiaucourt and Bolske 1996; Thiaucourt et al. 1996; Wesonga et al. 1998; AU-IBAR 2013; Atim et al. 2016; FAO 2017). This was inferred from outbreaks of CCPP following transport and mixing of apparently healthy animals with native ones (Hutcheon 1881; Perreau 1982). The duration of carrier state and/or latent infection in such survivors is not known. However, it may last for a quite long time may be upto 7 weeks (Hutcheon 1881; EFSA Panel et al. 2017). Chronic carriers can overcome the normal incubation period of 45 days (OIE 2017). Persistence of infection in an animal may be determined by the immune status of the animal. Better immune response is reflected in quicker expulsion. However, neither the actual duration of acute stage nor the persistence of infectivity is known. Consequently, it is still unclear if sterile immunity is reached by infected animals. Antibodies may help in timely removal of Mccp. Maternal antibodies show effect upto 8 weeks (King 1988) whereas innate antibodies persist longer, may be more than a year (Thiaucourt et al. 1994). The latent period is also variable and in an *in silico* model has been noted upto 7 days (Lignereux et al. 2018). Persistence in animals may be due to the inability to create an adequate immune response or the ability of the pathogen to survive without undesirable effects on the host. Age and health status may also affect this persistence period with young kids in poor health status being dominated by Mccp, whereas in healthy animals Mccp has the capability to survive without animals manifesting any clinical sign. This susceptibility feature is variable among different breeds of goats (EFSA AHAW Panel et al. 2017) with exotic breeds being more susceptible to clinical disease and native ones becoming susceptible only under any adverse stress such as environmental or managemental stress. Healthy Pashmina goats transferred from Ladakh to Kashmir valley, India developed clinical signs and lesions of the disease (Unpublished data). Alpine goats developed clinical disease whereas the local goats of Tunisia were not affected (Perreau 1982). Similarly, while signs were not shown by the angora goats of Turkey during the travel period of 7 weeks, they did when CCPP was exhibited in South Africa following the journey (Hutcheon 1881). Mccp can persist in animals which recovered from the disease but without becoming bacteriologically sterile and thus serve as chronic, latent carriers and hence considered as the cause for persistence of the disease (McMartin et al. 1980; Lefèvre et al. 1987b; Wesonga et al. 1993; Thiaucourt and Bolske 1996; Thiaucourt et al. 1996; Wesonga et al. 1998; OIE 2009; AU-IBAR 2013; Atim et al. 2016; Anonymous 2017). This was inferred from outbreaks of CCPP following transport and mixing of apparently healthy animals with the native ones (Hutcheon 1881; Perreau 1982). Further, certain antibiotics used for treating CCPP affected animals also result in carriers (El Hassan et al. 1984; Spickler 2015). Antibiotics may not prevent persistence in latent carriers (Thiaucourt et al. 1996).

**Figure 1.**
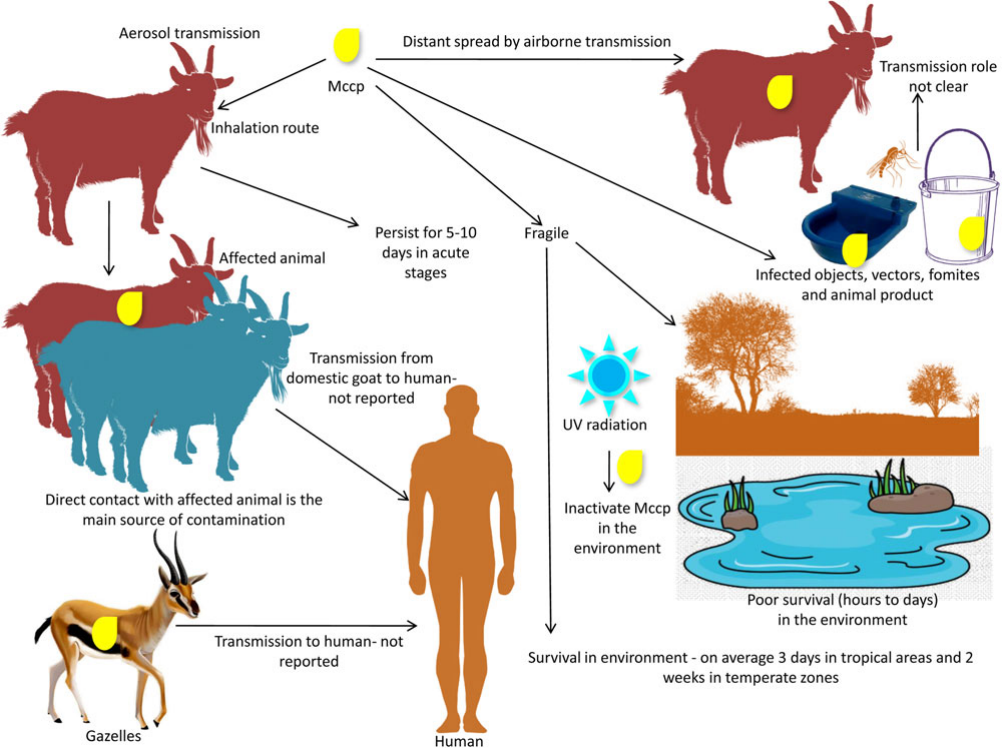
Transmission of *Mycoplasma capricolum* subsp. *capripneumoniae*.

The details of the transmission of Mccp are given in Figure 1.

### 2.6. Variability in prevalence studies

There is quite variability in the prevalence and incidence of CCPP, which is reflected by the local epidemiological conditions and the diagnostic tests employed given that some areas are endemic and some enzootic or under epidemics, and some diagnostic tests are more sensitive and specific than others. In enzootic areas, location of area coupled with diagnostic tests affect serological surveys and may predict different prevalence rates (Peyraud et al. 2014). Overall seroprevalence of CCPP was found to be 16% by Mekuria et al. (2008) using serological tests (CFT). Meta-analysis study by Asmare et al. (2016) in Ethiopia showed pooled prevalence of 39% in abattoir samples and 22% field samples giving net pooled prevalence of 26%. About 35–52% seroprevalence in goats and 23–36% in sheep was reported in Southern Tanzania (Mbyuzi et al. 2014), suggesting variability with species of animal. In eastern Turkey, Mccp has been isolated in 10 out of 24 herds equivalent to 38% with goats showing variable prevalence in different geographical regions (Cetinkaya et al. 2009). In India, some reports of prevalence from different states reflect a range of CCPP prevalence from 5% (Ramdeva et al. 2008) to 64% (Ghosh et al. 1989). For non-descriptive mycoplasmoses, a prevalence of 5% (Srivastava and Singh 2000) to 8% (Jain et al. 2015) has been reported. Other studies on prevalence are reported by Suryawanshi et al. (2015) in Maharashtra, India, Kumar et al. (2011) in Gujarat, India, Abraham et al. (2015) in one private goat farm, Thiruvananthapuram district, Kerala, India, Priya et al. (2008) in Wayanad Kerala, India and Srivastava et al. (2000) in Shimla, India, but none of these studies have given prevalence data. Recently we reported a seroprevalence of about 10% in Pashmina goats from Jammu and Kashmir State, India (Parray et al. 2019). From these studies, it can be inferred that prevalence varies from region to region, the test used for screening and the species of animal. However, even within the same country, variation in prevalence is noted indicating variable status of CCPP affected goats in different locations.

In most countries, meagre data on CCPP prevalence have been produced and where available figures are diverse. One of the reasons for variability is type of diagnostic test used and laboratory protocol followed. Time of observation also affects prevalence outcome of the study for a particular area. Frequent infection of goats with other mycoplasmas of the *Mycoplasma mycoides* cluster or peste des petits ruminants (PPR) or pasteurellosis infection to which CCPP is often confused in many parts of the world also cause variability.

Risk factors may also affect prevalence (Kipronoh et al. 2016; Parray et al. 2019). In our observation in Changthang Ladakh, India prevalence, based on survey, clinical and pathological observations, usually ranged from 5 to 40% with higher number of positive cases (upto 40%) or even outbreaks (10%) occurring during winter months especially in Rupshu (40%), Kharnak (38%), Samad (35%), Korzok (33%), Tsaga (39%), Nyoma (15%), Nidder (40%), Mudh (28%), Chushul (38%), Karygam (36%), Parma (35%), Satho (31%), Demchok (39%), Koyul (36%), Chumur (40%) villages over the years 2013–2017. During the summer months, usually only occasional cases were observed with no or sporadic outbreaks.

PCR, which is considered now as a routine diagnostic method for CCPP identification, is rarely practiced due to technical reasons and financial bindings. Similarly, novel cELISA for CCPP is also rarely available and not routinely used due to financial constraints and non-availability in various countries (EFSA AHAW Panel et al. 2017). Nevertheless, novel diagnostic methods like PCR have been used for studying molecular prevalence. Awan et al. (2010) reported for the first time the (molecular) prevalence of CCPP based on PCR in goats in Balochistan, Pakistan as 18% for Mccp. Similarly, in Saudi Arabia 29% positivity, 55 Mccp isolates out of 190 pleural fluid samples of CCPP, was noted by El-Deeb et al. (2017) also using specific PCR. Hussain et al. (2012) used counter immunoelectrophoresis (CIE) technique for studying seroprevalence of CCPP and found overall seroprevalence of 33% in Beetal goats in Pakistan. Swai et al. (2013) used monoclonal antibody-based cELISA and noted 3 and 32% overall animal herd- and village-level seroprevalence in Tanzania, respectively. Prevalence of CCPP in the Afar region in Ethiopia was only 15% on cELISA method, and in Narok region it ranged from 6 to 90%; in the Borana pastoral areas of Ethiopia 10% of goats showed pathological lesions of CCPP whereas the prevalence rate was 13% (Gelagay et al. 2007). Competitive ELISA was utilised also by Peyraud et al. (2014) for seroprevalence of CCPP in a global study and noted 15% seroprevalence in the Afar region of Ethiopia, 6–90% in Kenya, 15% in Mauritius, and 3–44% in northern Pakistan.

### 2.7. Morbidity rates

The morbidity rates represent the percentage of diseased animals and can be as high as 100% illustrating the highly contagiousness of the disease (MacOwan 1976; MacOwan and Minette 1976). In field cases, a morbidity of 80–100% is quoted (Rurangirwa and McGuire 1996), whereas in experimental infections, usually all the goats develop disease equivalent to about 100% morbidity (MacOwan and Minette 1978; Wesonga et al. 2004). However, in field outbreaks there are two perspectives: if the area is endemic, then CCPP develops usually only in naive animals, and if the area is free of disease, then a large population can get affected after the introduction of the pathogen. This has been observed in CCPP outbreak of South Africa contracted from Turkey where morbidity was more than 80% (Hutcheon 1881). We have also observed severe CCPP outbreak in Pashmina goats following transport from Changthang, Ladakh to Sonmarg, Jammu and Kashmir, India. In Greece, morbidity due to the disease was 98% in 1933 (Stylianopoulos 1933) and in Eritrea, it reached 90% around 2000 (Houshaymi et al. 2002). This indicates morbidity is also variable depending on breed, previous exposure or endemicity of CCPP. Variation due to location and weather may also be there. Morbidity values that ranged from 5 to 30% at lower altitudes and 56 to 68% at higher altitude have been recorded. This might be due to cold weather at higher altitudes, which is a predisposing factor (Thomas 1873). We have also noted higher percentage of disease cases (30–40%) during colder (winter) months than warm months (summer) in Changthang, India. Even within the same country or region, variability may be in different herds also. In tropical parts of India morbidity of about 60–80% is noted whereas in temperate parts we noted around 30–40% morbidity which may have fallen due to endemicity over the years. In eastern Turkey, of the 24 herds in various areas, about 41% of animals showed respiratory distress (Cetinkaya et al. 2009). In wild animals also, morbidity is variable with recorded morbidity of 100% in wild goats and 83% in Nubian ibex (OIE 2017). So, in a nutshell morbidity is determined by naiveness, breed, status of animal, location, environment, and stress on animal.

### 2.8. Case fatality and mortality rates

Case fatality and mortality rates in CCPP vary with age, breed, location, endemicity, environment or health status. The disease usually is fatal in younger age group, exotic breeds and those under harsh climate or stress and those which may not receive any therapeutic intervention show higher case fatality and mortality. Peracute cases or acute outbreaks have very high case fatality rate (CFR), especially when no therapeutics are used. Mortality rate may be up to 90% and CFR ranges from 60 to 100% (MacOwan 1976; MacOwan and Minette 1976) but usually mortality is around 80% (Rurangirwa and McGuire 1996; OIE 2009). However, it can be as low as 9% (Hussain et al. 2012) or as high as 100% (EFSA AHAW Panel et al. 2017). This variability is also due to sample size taken for the study or number of cases affected by the disease outbreak, period and area of observation and the observer. In Algeria, Hutcheon (1881) noted mortality higher than 75% when Litamoi et al. (1989) noted more than 50% in Kenya. Previously, Thomas (1873) had also quoted similar range from 50 to 75% and thereafter Rurangirwa and McGuire (1996) from 60 to 80%. However, in Greece, it varied from 60 to 94% with variability from herd to herd (Stylianopoulos 1933). Differences in mortality among domestic and wild animals may also exist with often the species getting exposed for the first time showing higher mortality. Mortality of 82% in wild goats and 58% in Nubian ibex has been noted (OIE 2017). Recently, Lignereux et al. (2018) noted higher mortality of about 70% in affected sand gazelles. In some outbreaks or experimental studies, lower mortality can occur. In an outbreak in Beetal goats in Pakistan mortality of 9% was reported (Hussain et al. 2012). In Oman, lower mortality rates of nearly 10% were also noted (Nicholas and Churchward 2012). Some reports did not claim mortality due to CCPP (Wesonga et al. 2004). In our observation, we noted only 5–15% deaths among the clinically and pathologically affected Pashmina goats, which may be due to the frequent and timely use of antibiotics against CCPP in this part of the world. It is believed that the mortality progressively decreases in endemic herds. Furthermore, decline in mortality is expected due to availability of advanced and highly effective antibiotics.

### 2.9. Public health

CCPP is not a zoonotic disease (AU-IBAR 2013; EFSA AHAW Panel et al. 2017; OIE 2017). There is no known risk of human infection with Mccp (OIE 2017) and till date no human case of infection by Mccp from goats has been recorded (EFSA AHAW Panel et al. 2017) neither there is any evidence that humans are infected by Mccp (Spickler 2015). In our observation of natural close confinement between Pashmina nomadic herdsmen (Changpas) of Changthang, Ladakh, India and Pashmina goats, we also could not yield any case when the clinically affected animals were kept or reared in residential tents/hamlets.

## 3. Clinical signs

CCPP is characterized by clinical signs of respiratory system involvement aggravated by mycoplasma induced systemic serofibrinous and inflammatory cascade that affect the lower respiratory tract (including the lungs), pleura and pleural cavity, and associated organs (heart), sometimes the upper respiratory tract, and rarely the eyes, joints, udder, liver and kidneys. The disease may be manifested in peracute, acute or chronic forms. Severe forms of the disease like peracute and acute forms usually result when fully susceptible herds are exposed to the pathogen, whereas chronic forms occur in endemically affected areas. In peracute form, death is sudden usually within 24–72 h and by definition without premonitoring (respiratory) signs (MacOwan 1976; MacOwan and Minette 1976; Samiullah 2013). In less acute or chronic form, and mildly affected or comparatively resistant animals’ signs of severe fibrinous pleuropneumonia, characterised by anorexia, depression, dyspnea, high fever (41–44 °C), coughing, nasal discharge, lagging, lying down, thorax pain, loss of body condition and heavy morbidity (upto 100%) and mortality (80–100%) are common (MacOwan and Minette 1976; Rurangirwa and McGuire 1996; Radostitis et al. 2009; OIE 2014). Generally following pyrexia (40.3–41.1 °C) of about 2–3 days, respiratory signs become evident (Radostitis et al. 2009). In clinical cases, abdominal respiration with accelerated and painful (pleurodynia) respiratory movements, snoring or wheezing respiratory sounds, frequent and productive coughing is observed. In terminal stages, inability to move, usually recumbency and if standing, then with base wide and neck extended (OIE 2009; Hussain et al. 2012; Wang et al. 2014; Shah et al. 2017; Tharwat and Al-Sobayil 2017).

Other respiratory signs include inspiratory dyspnea accompanied by grunting and snoring due to inflammatory lesions or exudations. Cough is generally painful, moist and productive and exacerbates on exercise. Continuous nasal discharge, which is initially serofibrinous straw coloured exudate followed by thick mucoid or purulent and rust coloured, might be observed (Sadique et al. 2012a, b; Zinka et al. 2013; Wang et al. 2014). Fever induced anorexia is usual and in pregnant animals abortion is occasional (Sadique et al. 2012a; Zinka et al. 2013; Wang et al. 2014). Clinical signs of CCPP typically correlate to respiratory system affection and these signs are common in susceptible herds and are independent of age and sex (Shah et al. 2017). However, these clinical signs may vary from animal to animal in an outbreak and with species of animal, severity of the phase of the disease be it peracute, acute or chronic. Variablity and indistinctness of clinical signs pose a challenge to accurately tentatively diagnose the disease.

## 4. Pathogenesis and pathology

Grossly consolidation of lungs is the main finding (100%) in CCPP on pathology often unilateral, followed by alveolar exudation and pleural fluid accumulation (91%) and pleural adhesion (73%) when the microscopic lesions mainly comprise of septal peribronchiolar fibrosis (82%), along with fibrinous pleuritis (64%) and peribronchiolar cuffing of inflammatory mononuclear cells (55%) in pulmonary tissue (Hussain et al. 2012). Mccp usually causes acute pathologic changes in young and immunocompromised animals and chronic changes in healthy resistant animals (OIE 2017). Among microscopic findings, macrophages have been found as the main cells in alveolar exudates followed by neutrophils along with few pulmonary fibrin deposits. Other marked histopathological changes noted were alveolar fibrin deposits, septal and peribronchial fibrosis, fibrous granulation tissue in the form of large strands, chronic fibrous pleuritis, septal fibrosis, lymphoid nodules and follicles surrounding airways, mononuclear alveolitis, bronchointerstitial pneumonia and bronchial lymph nodes displaying lymphoid hyperplasia (Wesonga et al. 2004; Hussain et al. 2012; Sadique et al. 2012a; Sheikh et al. 2016). We have also noted similar microscopic histopathological lesions including emphysema or atelectasis of alveoli, thickening of interlobular septa, granulation tissue, inflammatory cell infiltration, proteinaceous material deposition in alveoli, which are characteristic of CCPP in affected Pashmina goats (Parray et al. 2019).

Mycoplasma are extracellular pathogens of mucous membranes, and it is believed that they may attach to epithelial cells (Nicolet 1996). Adhesion of pathogen to host cells favors colonization for setting up of infection. Metabolic activity of mycoplasma releases free radicals like hydrogen superoxide and super oxide radicals, which can damage cilia or the membranes of cells. These events may be related as in other mycoplasma species (Almagor et al. 1984; Razin et al. 1998; Tanaka et al. 2014). Capsules noted in some mycoplasmas may play an important role in this aspect, especially the galactan of Mccp. The role of other mycoplasmal structures, for example, biological membranes is yet to be elucidated in the pathogenesis of the disease. Though the activation of the host immune system by Mccp is yet to be elucidated, it has the strong support of playing an important role in the pathogenesis of CCPP (Nicolet 1996). Immune cell stimulation by antigens of Mccp can aggravate or suppress immunity. The mitogenic stimulation of host immune cells especially lymphocytes, their antiphagocytic activity, suppression of immunity and auto-immune phenomena by antigens are well studied. Following stimulation, production of inflammatory mediators like proinflammatory cytokines, such as the tumour necrosis factor alpha (TNF-α), interleukins and interferon γ by immune cells (macrophages and monocytes) due to interaction with mycoplasma (Sacchini et al. 2012; Totté et al. 2015) has been reported for some but is yet to be worked out for Mccp. Mycoplasmal structures are mainly involved in this interaction and cytokine production. Biological membranes of mycoplasmas including plasma membrane, and their components like lipoproteins and lipids are believed to induce cytokine secretion. However, this has not been elaborated for Mccp yet though for other mycoplasmas a mechanism considered different than the bacterial lipopolysaccharides are thought to be involved (Razin and Tully 1995; Tully and Razin, 1996; Nicolet 1996). In our preliminary studies, we have noted an increase in levels of proinflammatory cytokine TNF-α, increase in total oxidant status and associated decrease in total antioxidant status in CCPP affected Pashmina goats suggesting inflammatory reaction and oxidative stress. This entire pathological mechanism might be initiated by antigens of Mccp which need to be explored.

Furthermore, antigens of mollicutes may be divided into surface antigens and cytoplasmic antigens (Alexander and Kenny 1977; Erno 1983). Surface antigens may be membrane-bound or part of an extramembraneous microcapsule (Erno 1987). Surface antigens are glycolipids and proteins. Superantigens can stimulate autoimmune response in mycoplasmoses (Nicolet 1996). They may also activate plasminogen induced inflammatory cascade (Bower et al. 2003). Surface antigens including very immunogenic lipoproteins, show antigenic variation hence escape the immune system of the host. Cytoplasmic antigens may include proteins, enzymes or metabolic products. A number of immunogenic proteins (translation elongation factor G, translation elongation factor Ts, trigger factor) and enzymes like dehydrogenases (pyruvate dehydrogenase, dihydrolipoyl dehydrogenase), acetyltransferases (dihydrolipoamide acetyltransferase, phosphate acetyltransferase, adenine phopshoribosyl transferase), reductases (FMN-dependent NADH-azoreductase, peptide methionine sulfoxide reductase), phosphopyruvate hydratase, transketolase and inorganic diphosphatase have been noted in other mycoplasma subsp. (*M. mycoides* subsp*. capri*) (Corona et al. 2013), hence need to be explored for Mccp.

These studies reveal an array of wide spread pathological changes whose molecular basis needs to be explored. The pathogenesis of CCPP may involve inhalation, attachment, ciliostasis, the alteration and loss of cilia, multiplication, and destruction of the mucosal epithelial cells, dissemination and inflammation and oxidative stress which is usual in mycoplasma infections but has not been studied for Mccp yet. After entry into respiratory passages, the Mccp may attach to superficial cell layers by different membrane structures. This follows colonization and initiation of pathological inflammation, which is characterized by ciliostasis of epithelia, serofibrinous pleuropneumonia, vasculitis, and fibrinocellular exudation. Mycoplasmal antigens (polysaccharides, galactan, lipoprotein) activate the immune system and stimulate the inflammatory and oxidative cascade (Darzi et al. 1998; Chambaud et al. 1999; Maritim et al. 2018), resulting in pathogenic changes and hence wide spread sero-fibrinous inflammatory reaction and fluid exudation especially in respiratory system organs involving lungs, pleura, thorax attachments, and sometimes heart, liver, and kidneys. These pathological alterations grossly lead to fibrin deposition in the pleural cavity, fluid exudation and hydrothorax, which are the principle pathological lesions of the disease. CCPP is histopathologically characterised by interstitial pneumonia of the pulmonary tissue along with interstitial and intralobular oedema (Kaliner and MacOwan 1976).

**Figure 2.**
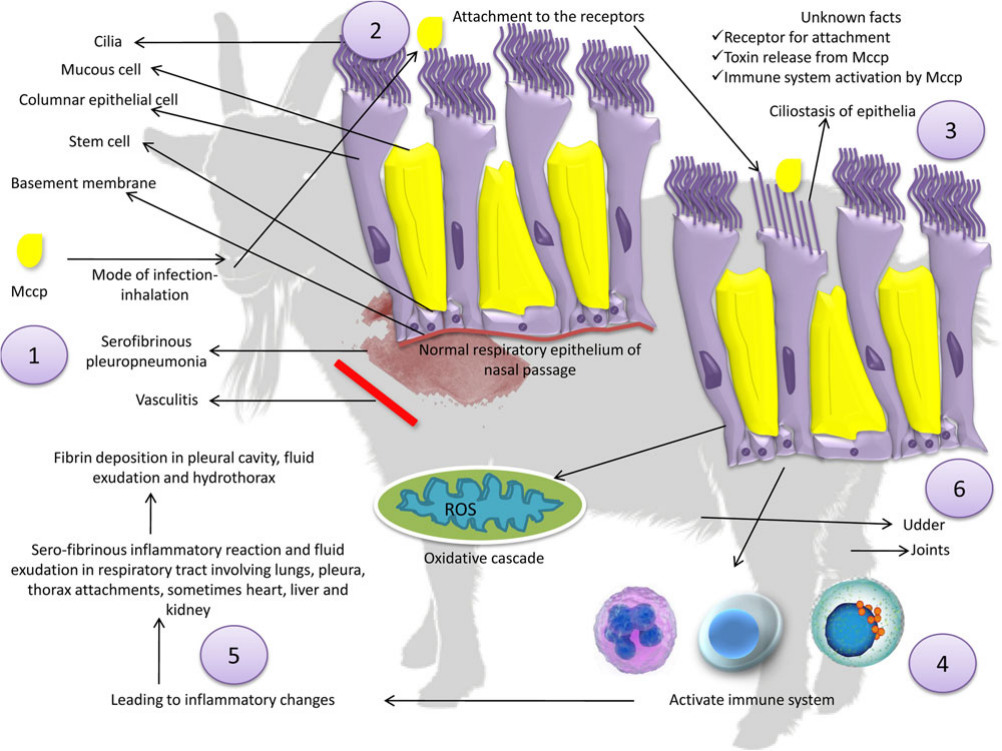
Pathogenesis of *Mycoplasma capricolum* subsp. *capripneumoniae*. (1) Inhalation is the commonest route of infection through aerosol transmission, (2) colonization, (3) ciliostasis of epithelia, serofibrinous pleuropneumonia, vasculitis, and fibrinocellular exudation, (4) Mycoplasmal antigens (polysaccharides, galactan, lipoprotein) activate immune system, (5) stimulation of inflammatory and oxidative cascade, (6) *Mycoplasma capricolum* subsp. *capripneumoniae* may also affect other organs like joints, eyes, and udder.

Keeping in view, all the above-mentioned studies the molecular basis of pathogenesis specifically pertaining to Mccp need to be further explored. The pathogenesis of Mccp has been detailed in Figure 2.

## 5. Diagnosis

Diagnosis is one of the most important and challenging aspects of the disease as it influences prophylactic and therapeutic regimen and the control strategies for prevention of global spread. Diagnosis might involve microbiological, biochemical, serological, and gene-based identification following a clinical tentative diagnosis. Microbiological methods include culture, isolation, and identification, which are rather conventional ones but are still considered as standard methods of detection of Mccp. However, the microbiological diagnosis of CCPP is considered difficult for two main reasons, the first being very poor *in vitro* growth of Mccp, and secondly, usual contamination of samples by other easily growing mycoplasmas (Thiaucourt et al. 1996). In addition, fastidiousness and special requirements of Mccp add to the problem of diagnostics. Hence, other diagnostic methods should be relied on (Thiaucourt et al. 1996).

All these tests are either based on the growth and metabolic characteristics of Mccp, for example, ability to ferment glucose, digest casein and serum, reduce tetrazolium (Adehan et al. 2006; Soayfane et al. 2018) or to its immunogenic potential, for example, surface antigens which are glycolipids and proteins, as detected by CFT and ELISA, respectively (OIE 2014; Peyraud et al. 2014), and genetic make up, specific genes or loci, for example, 16S rRNA genes (Bascunana et al. 1994; Bolske et al. 1996), CAP-21 genomic regions (Hotzel et al. 1996), 316 bp long sequence of arginine deiminase (ADI) (Woubit et al. 2004), or H2 locus (Lorenzon et al. 2002; Manso-Silván et al. 2011) as detected by PCR-based systems. For these diagnostic methods, specific sample collection, transportion in aseptic and standardized condition is essential for the accuracy of the diagnostic method.

### 5.1. Sampling

Appropriate sampling is an important aspect for accurate diagnosis of the disease. Sample size, type, and preparation depend on availability, facilities and local feasibility. As many as possible samples should be collected under standard procedures (OIE 2014) within an aseptic environment. Nasal swabs, pleural fluid and lung samples (from necropsied animals) are collected from goats showing typical signs of CCPP. Blood or serum samples are essential for serology and discharges, exudates, blood and tissues for culture or isolation and gene/DNA-based studies. Nasal swabs are collected after proper cleaning of the external nares and are placed in universal transport media. Lung samples are aseptically collected at necropsy from the interface between affected and nonaffected parts and kept in sterile disposable plastic bags. Pleural fluids are collected by sterile syringes. Thoracocentesis or bronchoalveolar lavage (BAL) is also preferred to minimize contamination. Thoracocentesis is done usually at the seventh intercostal space on left thorax or sixth intercostal space on right thorax keeping needle/cannula in a cranial direction to prevent intercostal vessel or nerve damage on caudal end of the rib. Usually, hair is clipped, skin is anesthetized by local anesthesia after proper disinfection. Another desirable method is lower intrathoracic puncture with the help of broad diameter needles and USG guidance. About 10 ml of pleural fluid is sufficient. Samples of hepatized pulmonary tissue are also suitable. Three square centimeter sections need to be taken from affected lung tissue near normal healthy tissue (Thiaucourt et al. 1996). All samples are transported to the laboratory and cooled in an icebox (at 4 °C), if requires longer time (days to weeks) then it can be frozen at −20 °C, for longer duration (months) samples can be stored at −70 °C with antibiotics as penicillin or ampicillin added to prevent contamination (Thiaucourt et al. 1996; OIE 2014; El-Deeb et al. 2017). Blood samples can be collected by jugular venipuncture and serum can be harvested and stored at 4 °C. Tissue samples need to be properly processed before further investigation. Proper processing of lung samples is essential for accurate histopathology and isolation of organisms. The surface of each lung tissue with the lesions is sterilized with a hot spatula and deep tissue is minced with sterilized scissors. One gram of minced tissue is mixed with 9 ml of modified pleuropneumonia-like organisms (PPLO) medium (OIE 2014; El-Deeb et al. 2017) and stored for further analysis.

### 5.2. Isolation and identification

Isolation of Mccp is considered as a confirmatory diagnosis but is a difficult task and requires technical expertise for proper isolation and identification (Bolske et al. 1996). This being a tedious job not only because of the practical difficulty of obtaining appropriate samples but also of the fragile and fastidious nature of the pathogen (Thiaucourt et al. 1996). Besides difficult isolation and highly technical expertise, Mccp requires a very special growth medium, well-equipped and sophisticated laboratory facility as the pathogen is very fastidious and requires a prolonged initial incubation for at least 5–7 days (average 4 days) at 37 °C with upto 5% carbon dioxide under sterile laboratory environment (Freundt et al. 1983; Thiaucourt et al. 1996; Awan et al. 2010; OIE 2014). Sampling, culture, and isolation are done as per standard procedures (OIE 2014) or depending on regional modifications (Thiaucourt et al. 1996; Awan et al. 2010; Kumar et al. 2011; Sadique et al. 2012c; El-Deeb et al. 2017). Nasal discharges, swabs, pleural fluid, lung tissue or pleural tissue are appropriate samples for isolation (Freundt et al. 1983; Thiaucourt et al. 1996; Awan et al. 2010; OIE 2014). Media, liquid broth or solid agar either readymade or prepared in laboratory generally contains nutrients or supplements that can support amino acids, nucleotides and lipid synthesis as Mccp lacks the capability of synthesizing these essential nutrients. Media usually contains beef heart, infusion (6.0 g), peptone (10.0 g), sodium chloride (5.0 g), agar (14.0 g) and supplemented by yeast extract (0.09 g) and horse serum (22.8 ml).

Media for isolation of organisms can also be prepared from beef heart, infusion from solids (2.0 g), pancreatic digest of casein (7.0 g), beef extract (3.0 g), yeast extract (3.0 g), sodium chloride (5.0 g), agar (14.0 g) enriched by horse serum (20.0 ml), yeast extract (fresh autolysate) (10.0 ml) and thallium acetate (50.0 mg). Such media are generally supplied commercially.

Generally, nasal swabs but preferably pleural fluid, which contains maximum concentration of mycoplasmas, need to be collected and directly cultivated on PPLO agar or broth media under aseptic procedures (MacOwan and Minette 1978; Bolske et al. 1996; Thiaucourt et al. 1996; Bashiruddin and Windsor 1998; Noah et al. 2011; El-Deeb et al. 2017). Then inoculated plates or tubes are incubated at 37 °C in a humid atmosphere of 5% CO_2_, sometimes with 95% N_2_ (Thiaucourt et al. 1996; Noah et al. 2011; El-Deeb et al. 2017). Aseptically collected pleural fluid, or minced tissue suspension (after vigorous shaking) are 10-fold serially diluted up to 10^4^ in PPLO broth, then filtrated through appropriate filter usually 0.45 μm pore size and incubated at 37 °C. Each dilution is also plated on PPLO agar and incubated at 37 °C in a humid atmosphere of 5% CO_2_ and 95% N_2_. Liquid media are observed daily or every third day for the evidence of growth (change of pH indicated by a color change and appearance of floccular material) for up to 10–14 days (MacOwan and Minette 1978; Bolske et al. 1996; Thiaucourt et al. 1996; Bashiruddin and Windsor 1998; Noah et al. 2011; El-Deeb et al. 2017). Tubes with positive growth are subcultured on PPLO agar and incubated as before. Tubes which show no signs of growth are discarded after 10–14 days’ incubation. Plates are inspected every 3 days using stereomicroscope (4× magnification) for the presence of characteristic mycoplasma colonies (MacOwan and Minette 1978; Bolske et al. 1996; Thiaucourt et al. 1996; Bashiruddin and Windsor 1998; Noah et al. 2011; El-Deeb et al. 2017). Plates which show no mycoplasmal growth are discarded after 14 days. Purification and cloning of primary culture are performed by transferring of a small agar block bearing isolated colonies of mycoplasma to PPLO broth and incubated at 37 °C for 3–5 days. Isolates are permissively identified based on colony morphology, staining with Gram staining, Giemsa or Diene’s stain or biochemical tests (Gupta 2005; Sadique et al. 2012c; Shah et al. 2017).

Following isolation, identification of the colonies or the organism is an important diagnostic aspect. Usually identification is made by the appearance of characteristic colony types grown on solid agar-based medium (Srivastava et al. 2000; Mondal et al. 2004) when the turbidity of broth medium is less reliable, a characteristic yellow turbidity of broth media develops after 3 or more days. Mycoplasma colonies are typically characterized by fried egg-like appearance, often small, tiny, and smooth with a diameter of about 1–2 mm, and dense elevated centres embedded in the medium. Though Mccp colonies have similar characteristics, in practical observation they may vary, especially during initial culturing process (Parray et al. 2019). The characteristics of the colonies depend on media used, species of mycoplasma, its passage level and the age of the culture (OIE 2017). In early passage, colonies are usually of irregular morphology, usually small, centreless, and of non-uniform bizarre shape. These isolates produce characteristic ‘fried egg’ colonies after passage (OIE 2017). Identification of isolated mycoplasma in the initial stage is done by conventional (Gram staining) and specific staining (Diene’s stain) or by serological tests, growth inhibition (GI) test or biochemical tests (Cluss and Somerson 1984; Sadique et al. 2012c; Shah et al. 2017) and potential other available molecular diagnostics like nucleic acid amplification techniques (NAATs) that encompass a variety of chemistries used for detection, to more sophisticated characterizing methods such as multi-locus variable-number tandem-repeat analysis (MLVA), multi-locus sequence typing (MLST), matrix-assisted laser desorption ionization-time-of-flight mass spectrometry (MALDI-TOF MS), among others (Diaz and Winchell 2016).

Among the tests available the simplest and most specific is considered to be the GI test, but its low sensitivity makes it less reliable. This test uses specific hyperimmune serum for inhibition of mycoplasma growth on solid agar media. Primary surface antigens of Mccp are also detected (Dighero et al. 1970). It is especially helpful for identifying the pathogen as they are serologically homogeneous and large inhibition zones are produced for various isolates. Despite monoclonal antibody being used for preventing cross-reaction between Mccp and *Mycoplasma capricolum* subsp. *capricolum* (Rurangirwa et al. 1987b), cross-reactions may nevertheless occur (Belton et al. 1994). Genetic resemblance among mycoplasmas renders GI tests less definitive (Awan et al. 2010).

Mccp is able to ferment glucose, digest casein and serum, and reduce tetrazolium, but could not hydrolyse arginine (Shah et al. 2017). However, classical biochemical tests are not considered definitive for identification of Mccp because of their genetic resemblance with other members (Awan et al. 2010). Biochemical characterization of the isolates is performed and it includes glucose hydrolysis, reduction of tetrazolium chloride, phosphatase activity, hydrolysis of arginine, film and spot formation, serum digestion, and digitonin sensitivity (Aluotto et al. 1970; Gupta 2005; Awan et al. 2010; Kumar et al. 2011; El-Deeb et al. 2017). Adehan et al. (2006) noted that Mccp hydrolysed glucose, reduced tetrazolium chloride, and weakly digested serum. Recently, based on oxygen uptake rates, Mccp have been divided into two major biochemical groups, namely organic acid-oxidizing group that metabolise oxidised organic acids and glycerol and the glucose-oxiding group that metabolises sugars (Soayfane et al. 2018). These were well illustrated by DNA-DNA hybridization tests as different species (Soayfane et al. 2018).

### 5.3. Haemato-biochemical parameters

Hematological changes were studied by Abdelsalam et al. (1988) in CCPP-affected goats. Usually, in field cases, hematological findings are not so much relevant to the diagnosis of the disease, but anemia and leukocytosis followed by leucopenia have been noted in animals affected with mycoplasmosis (Mondal et al. 2004). Lower blood total protein, albumin and elevated ASAT, ALAT, calcium, glucose, and globulin have been reported in mycoplasma affected goats in comparison to control ones (Mondal et al. 2004).

### 5.4. Sero-molecular tests

Molecular tests have become the novel diagnostic interventions for CCPP not only because of high specificity and sensitivity, but also because of difficulties in culturing of Mccp. Globally, various molecular tests are being employed for diagnosis of CCPP. Broadly they are serological and amplification-based tests, and their diagnostic capability depends on antigens, antibody, and genes/DNA either of Mccp or against it (antibody). Agglutination tests (non-specific and specific), immunoassays (enzyme-linked immunosorbent assay [ELISA]), fluorescent antibody assay (FAT), complement fixation assay (CFT), passive or indirect haemagglutination test (IHT), amplification tests (PCR, hybridization, sequencing) are the currently employed diagnostic tests with latex agglutination, cELISA, and PCR being of prime importance and routinely used in global studies. However, the field level availability or even absence in research facilities in most of the countries is a major limitation. Moreover, cost involvement is another binding on use in limited spheres (Liljander et al. 2015). Nevertheless, novel sensitive and specific, field applicable and cost-effective versions of these diagnostic tests are utmost important and focused on for development.

Non-specific agglutination tests have been developed (Rurangirwa et al. 1987; Srivastava and Singh 2000), but it cannot differentiate from other species and subspecies of mycoplasma especially among mycoides clusters (Bertin et al. 2015). Specific latex agglutination tests (LAT) against CCPP have been utilized for seroepidemiological studies but cross-reactions are a cause of concern as it is based on capsular polysaccharide or antigen specific to it (Rurangirwa et al. 1987; March et al. 2000; OIE 2014; Bertin et al. 2015). ELISA has been utilized for quite some time for elucidating the seroepidemiology with cELISA being the novel test recommended with high specificity (99.8–100%) (Thiaucourt et al. 1994; Mbyuzi et al. 2014; OIE 2014; Peyraud et al. 2014; AU-IBAR 2015; Asmare et al. 2016; EFSA AHAW Panel et al. 2017). Though these serological tests have proven beneficial for diagnosing the disease most preferably studying seroepidemiology, reflecting past infections with occasional limitations of cross-reactivity and low seroconversion. However, the current disease status and frequent outbreaks demand early, rapid and accurate diagnosis with high specificity and sensitivity along with less or no financial implications. For this, gene or DNA-based tests have been tried, and have also helped in the differentiation from other pathogens (Settypalli et al. 2016) in addition to members of mycoides cluster (Taylor et al. 1992; Thiaucourt et al. 1992; Bascunana et al. 1994; Bolske et al. 1996; Hotzel et al. 1996; Bashiruddin 1998; Woubit et al. 2004; Lorenzon et al. 2008; OIE 2014). PCR (Bascunana et al. 1994; Bolske et al. 1996; Hotzel et al. 1996; Woubit et al. 2004; Lorenzon et al. 2008; OIE 2014; Liljander et al. 2015), DNA probes (Taylor et al. 1992; Bonnet et al. 1993), hybridization (Maigre et al. 2008) and sequencing (Thiaucourt et al. 2000; Manso-Silván et al. 2011; Dupuy and Thiaucourt 2014; Falquet et al. 2014) have been used for better understanding of Mccp and CCPP diagnostic studies and are being revised from time to time. The other features of these tests include non-requirement of live organism, can be transported to sophisticated laboratory, small amount of DNA can suffice and higher specificity and sensitivity and hence are considered as confirmatory tests.

### 5.5. Serological tests

A number of studies on development of serological tests have been listed with only few been able to have field applications. However, a brief insight helps in overviewing these developments. Initial study on complement fixation test (CFT) was by MacOwan and Minette (1976) for diagnosing CCPP. Based on mycoplasmaemia and cross-protection tests in mice, Hooker et al. (1979) differentiated *Mycoplasma mycoides* subsp. *mycoides* from some mycoplasmas affecting goats. Passive haemagglutination test (PHT) and CFT were reported as diagnostic tests for CCPP by Muthomi and Rurangirwa (1983). These tests required laboratory set-up and were less sensitive hence not feasible at field level. For field diagnosis of the disease, LAT was reported to be ideal (Rurangirwa et al. 1987). Refinements in serological diagnostics lead to vast application of these tests for investigating CCPP in different countries. Serological studies on the disease in Oman were carried out by Jones and Wood (1988). Further improvement in serological diagnostic tests was in evaluating sensitivity. Litamoi et al. (1989) reported that the slide agglutination test (SAT) is more sensitive than complement fixation for detection of antibodies in vaccinated goats. However, these tests were non-specific hence for specificity ELISA was used for detecting antibodies to Mccp (Wamwayi et al. 1989). Lefèvre et al. (1993) mentioned different field diagnostic kits for CCPP including serological tests. As these serological tests too proved non-specific and cross-reaction by different mycoplasmas was its main drawback monoclonal antibodies were used by Thiaucourt et al. (1994) in the diagnosis of CCPP which were specific to Mccp. Specificity of a monoclonal antibody was studied by Belton et al. (1994) to *Mycoplasma capricolum* strain F38. Elaborating different diagnostic strategies for CCPP, Thiaucourt et al. (1996) in their review highlighted advantages and disadvantages of various diagnostic tests including serological tests. As previous specific serological diagnostic tests were laboratory oriented and difficult to use under field conditions exploration of specific and field applicable diagnostic tests was started. This was possible by evaluating highly Mccp-specific antigens. Thus, based on Mccp capsular polysaccharide-specific antigen a LAT was developed by March et al. (2000) for the rapid detection of CCPP. It is believed to be more sensitive than CFT and can detect 2 ng of capsular polysaccharide (1.7 × 10^4^ CFU) in 0.03 ml of serum. Simultaneously, serological investigations were carried out utilizing these serological tests in Ethiopia by Sharew et al. (2005). Further ahead in serodiagnostics, tests based on same antigens needed to be evaluated for specificity, sensitivity, field applicability and the overall improvement in diagnosis. Thus Wambugu (2005) developed an I-ELISA using a unique and specific capsular polysaccharide epitope to Mccp for better diagnosis than the field LAT of similar antigen basis. I-ELISA proved improvement in diagnosis with the high analytical and diagnostic specificity, objectivity and its capacity to run more samples than LAT. It is assumed to have facilitated seroepidemiological studies in developing countries. These serological tests were employed for studying seroprevalence of CCPP globally. Blocking-ELISA (B-ELISA) was used by Sharew et al. (2005). Mekuria et al. (2008) used CFT. Hussain et al. (2012) used CIE technique for studying seroprevalence of the disease. CFT though recommended as a designated test for international trade and one of the diagnostic tests for endemic regions but sensitivity and/or specificity of CFT for CCPP diagnosis is not known yet (Radostitis et al. 2009; OIE 2014; Asmare et al. 2016) and is believed to be low as it shows cross-reaction due to use of a crude antigen. Furthermore, about 80–100% animals experimentally infected with Mccp have not been detected by CFT (March et al. 2000). Hence, French Agricultural Research Centre for International Development (CIRAD) recently developed a competitive ELISA test for CCPP. This competitive ELISA kit for the disease is a modification of the blocking ELISA formatted at CIRAD (EFSA AHAW Panel et al. 2017) and has high specificity (99.8–100%) (OIE 2014; Asmare et al. 2016) as it is based on Mccp-specific monoclonal antibody (Thiaucourt et al. 1994). Using this cELISA recently in an international collaborative study, Peyraud et al. (2014) studied prevalence of the disease in some African, Asian and Middle Eastern countries. I-ELISA was used by Atim et al. (2016) and LAT by El-Manakhly and Tharwat (2016), Sheikh et al. (2016), and Tharwat and Al-Sobayil (2017). Bahir et al. (2017) recently comparatively evaluated LAT, I-ELISA and cELISA for studying seroprevalence of CCPP in eight provinces of Afghanistan and found that out of 325 samples, 53 tested positive by LAT, 64 by I-ELISA and 38 by cELISA. Similarly, we also evaluated SAT and cELISA for seroprevalence of CCPP in Pashmina goats and noted that of the 48 SAT positive serum samples 43 tested positive on cELISA (Parray et al. 2019). From these studies, it can be inferred that the LAT, ELISA and CFT are routinely used serodiagnostic tests with current forms of improved LAT being suitable for application in the field with comparable sensitivity and specificity to ELISA-based tests and better than CFT, CIE, and PHT. However, on laboratory platforms ELISA-based tests are usually more sensitive and specific than others.

However, the main limitations of these serological tests have been non-specificity (Bertin et al. 2015), requirement of sophisticated infrastructure, stationary laboratory and time of operation in addition to cost involvement (Rurangirwa and McGuire 1996; Liljander et al. 2015). Hence, recent focus has been on specificity, field applicability and cost-effectiveness.

### 5.6. DNA or gene-based molecular tests

Many of DNA- or gene-based molecular tests are proving beneficial and are considered as authentic in confirming the disease and identification of Mccp, which was until recently, solely dependent on the highly cumbersome and difficult culture and isolation of Mccp for confirmation of the presence of CCPP. Besides, culture and isolation based on hectic diagnosis, serological tests are also not specific for diagnosis process. Hence, novel DNA probes-based diagnostics have augmented diagnosis. They include ribosomal ribonucleic acids genes (cloned ribosomal RNA genes), complementary deoxyribonucleic acid to mycoplasmal ribosomal ribonucleic acids (cDNA to mycoplasmal rRNA), synthetic 16S rRNA oligonucleotide sequences, or cloned mycoplasmal protein genes (Razin 1994). The DNA probe developed by Taylor et al. (1992) helped in differentiating Mccp from the *M. mycoides* cluster members. PCR-based diagnostic systems have emerged as promising tools for the early and rapid detection, specific identification and proper differentiation of members of the *M. mycoides* cluster especially for the specific identification of Mccp (Bashiruddin et al. 1994; Hotzel et al. 1996). *Mycoplasma mycoides* cluster members have some unique features of genome, for example, two rRNA operons (Christiansen and Ern⊘ 1990). These rRNA operons differentiate them from other mycoplasmas. However even within the cluster different genes have been identified in some members, for example, 16S rRNA genes of the two operons in Mccp which help in identification of these members among cluster. Hence, PCR systems have been developed based on the sequence of the gene for 16S ribosomal RNA (Bascunana et al. 1994) wherein final identification of Mccp is done following restriction enzyme analysis (REA) test by restriction enzyme Pst1 (Bolske et al. 1996). Thus, PCR followed by REA with Pst1 has been considered as diagnostic for Mccp (Christiansen and Ern⊘ 1990; Johansson et al. 1994; Bolske et al. 1996; Pettersson et al. 1998). This system is applicable to clinical material including field samples like pleural fluid, nasal, pharyngeal, ear discharge and lung tissue.

Thiaucourt et al. (2000) used sequencing of a putative membrane protein gene for the phylogeny of the *Mycoplasma mycoides* cluster and noted that Mccp can be easily identified by three specific nucleotide positions or by sequencing the 1298 bp long fragment. Improvising PCR techniques for convenience and identifying novel and specific DNA segments lead to emergence of new PCR protocols for Mccp. Another specific PCR system was developed by Woubit et al. (2004) for Mccp. This system detected arcD gene as a differentiating DNA fragment for Mccp. Previous PCR-based tests were qualitative for the detection of Mccp. However, Q-PCR was developed by Lorenzon et al. (2008). It was a specific real-time PCR assay for the detection and quantification of Mccp besides it was a rapid and sensitive test. Similarly, Fitzmaurice et al. (2008) standardized RT-PCR assays for the detection of members of the *Mycoplasma mycoides* cluster including Mccp. Sensitivity and specificity raised few concerns about PCR systems besides applicability on tissues. Hence, loop-mediated isothermal amplification (LAMP) was used by He et al. (2014) for sensitive and rapid detection of Mccp exploring H2 gene sequences of Mccp. It was a sensitive and specific test for Mccp and able to detect Mccp in tissue.

As a result, these PCR-based diagnostic systems proved effective both at field and laboratory level diagnosis. However, with passage of time need for exploring full genome of Mccp and strain-specific differentiation among Mccp strains arose. Chu et al. (2011) studied genome sequence of Mccp strain M1601. Manso-Silván et al. (2011) studied multi-locus sequence analysis of Mccp. This also helped in molecular epidemiology of CCPP. Dupuy and Thiaucourt (2014) studied complete genome sequence of Mccp strain 9231, whereas Abomsa and Falquet et al. (2014) studied complete genome sequence of Mccp strains F38 and ILRI181. For the fine differentiations of strains, an extended multilocus sequence analysis (MLSA) technique was employed by Dupuy et al. (2015). By this technique, a total of six clades were identified within Mccp strains indicating different regions, namely East Africa, Central Africa, Central Asia, East Asia, Arabian Peninsula, and Mediterranean Basin. As other respiratory diseases (PPR, pasteurellosis) complicate the diagnosis of CCPP besides frequent occurrence of other common diseases in goats (goat pox) necessitated devising a diagnostic test that can simultaneously and accurately diagnose all these diseases. Thus, a multiplex PCR was developed by Settypalli et al. (2016) which helps in simultaneous detection of mycoplasma (Mccp), viruses (PPR virus, capripoxvirus) and bacteria (*Pasteurella multocida*).

Thus, the molecular gene-based techniques including PCR, DNA hybridization, and sequence analysis can become the tools of choice for evaluating phylogenic relationships among mycoides cluster (Taylor et al. 1992; Bashiruddin 1998). They have confirmed the closeness of these members as was previously believed besides elucidating differences among the members (Costas et al. 1987; Bashiruddin et al. 1994; Bashiruddin 1998), and thus resulting in rapid diagnosis through detection and identification based on DNA amplification by PCR and restriction fragment length polymorphism (RFLP) (Costas et al. 1987; Bashiruddin et al. 1994; Bashiruddin 1998). These tests have found field applicability and utility worldwide.

Despite revitalizing molecular diagnosis by PCR systems, the field applicability always remained a cause of concern in addition to requirements of sophisticated laboratory equipment and technical personnel. However, Liljander et al. (2015) developed a field-based RPA for rapid detection of Mccp. Besides being rapid, it was quite specific and sensitive assay utilizing isothermal DNA amplification with recombinase polymerase amplification. It is applicable directly to CCPP positive animals and does not require prior DNA extraction, sophisticated laboratory or technical personnel. Detection limits are reported to be 5 × 10^3^ cells/ml for genomic DNA and 5 × 10^4^ cells/ml for culture and the detection signal is produced within 15–20 min.

In our study on 433 Pashmina goats we evaluated SAT, cELISA and PCR-RFLP for diagnostic and epidemiological purposes and of 48 serum samples tested positive on SAT, 43 on cELISA and 42 pleural fluid/nasal discharge samples tested positive on PCR-RFLP proving PCR and cELISA are more sensitive and specific than SAT (Parray et al. 2019). Thus, PCR has been used as an extensive diagnostic tool for Mccp (Bolske et al. 1996; Thiaucourt et al. 1996; Thiaucourt and Bolske 1996; Monnerat et al. 1999; March et al. 2000; Shankster et al. 2002; Peyraud et al. 2003; Woubit et al. 2004; Waleed et al. 2006; Maigre et al. 2008; Awan et al. 2010; Kumar et al. 2011; Wang et al. 2014; Ejaz et al. 2015; El-Deeb et al. 2017; Saeed and Osman 2018). However, further exploration of highly specific loci will enable proper and highly specific detection and hence definite confirmation of CCPP besides devising field applicable and cost-effective techniques.

### 5.7. Diagnostic imaging (USG and X-ray)

USG has been a recent advanced tool for diagnosing CCPP-associated changes in lungs, pleura, thorax, and associated structures. Irregularity of the visceral pleural surface, which reflects the beginning of consolidation can be revealed by USG. Accumulation of exudates makes alveoli hypoechoic or even anechoic in pleuropneumonia, which otherwise form hyperechoic zones both in normal or consolidated lungs. However, with the passage of consolidation, a reduction of these hyperechoic zones occurs, and hyperechoic reflective bands may appear. Consolidated area appearing hypoechoic and homogenous reflects exudate, blood or mucous accumulation. Anechoic area between lung, thorax wall, diaphragm and heart indicate pleural effusion along with deep acoustic enhancement. Increase in the gap between parietal and visceral pleura due to fluid accumulation can also be detected by USG. Thickened septa may show increased echogenicity. USG is not able to visualize physiologically normal lung as air does not allow passing of waves but a diseased lung or a number of respiratory pathologies which alter air and fluid level, or membrane structures can be detected (Braun et al. 1997; Tharwat and Oikawa 2011; Tharwat and Al-Sobayil 2017).

Recently, Tharwat and Al-Sobayil (2017) studied ultrasonographic changes in goats affected with CCPP. They used USG both as diagnostic and post-mortem tool. They quoted that sonography can be helpful in detecting extension and severity of changes in affected lung, thus favoring prognosis. Liver-like echotexture, unilateral hypoechoic pleural effusion and turbid or greenish pleural fluid exudation, hypoechoic or anechoic area surrounded by hyperechoic zone indicating pleural abscessiation, echogenic tags immersed in the pleural fluid indicating fibrin deposits, extended echogenic areas with immersed anechoic areas indicating fibrin adhesions with exudate filled pockets, hypoechoic pericardial areas with echogenic tags overlying the epicardium indicating pericardial effusions and fibrin deposition on epicardium were some of the USG findings revealed by Tharwat and Al-Sobayil (2017) in CCPP affected goats.

X-ray findings reflect consolidated areas of lung as dense and white areas. If the larger airways like bronchi or even alveoli are not affected, they are of relatively low density (blacker). This creates air bronchogram which are characteristic sign of consolidation. Other X-ray findings in consolidated lungs are poorly defined uniform or homogeneous opacity obscuring blood vessels, formation of silhouette, loss of lung or soft tissue areas or interface, air-bronchogram extension to the pleura or fissure, but usually not crossing it, and no volume loss. Mycoplasmal pneumonia starts perihilar and can become confluent and/or patchy and no air bronchograms as disease progresses. On X-ray there is opacity with pleural effusion, airspace opacity, lobar consolidation, or interstitial opacities.

## 6. Treatment

Several antibiotics have been used against Mccp for the treatment of CCPP (Onovarian 1974; El Hassan et al. 1984; Yatoo and Kanwar 2016; Yatoo et al. 2018). However, macrolides especially tylosin is considered to be the drug of choice against Mccp. Tylosin is usually rarely available in the areas where CCPP is prevalent. The use of oxytetracycline against CCPP has proven effective and has been used for quite long time but usage over long period in most of the areas can predispose to side effects (teratogenicity in goat kids) besides the risk of antibiotic resistance with additional risk being imposed by prophylactic usage that too in minimal doses enabling mycoplasma to better cope up the antibiotics. Though other antibiotics like fluoroquinolone (enrofloxacin, danofloxacin), aminoglycosides (streptomycin), and pleuromutilin (tiamulin) are being used and newer ones among these classes being explored against Mccp but cost, availability, acting period and convenience in use and creation of carrier state are major determinants in the application of antibiotics in CCPP prevalent areas. Earlier combination of dihydrostreptomycin sulphate with penicillin G procaine was used for treatment of CCPP at a dose rate of 30 mg/kg BW for each intramuscularly (Rurangirwa et al. 1981a). Dihydrostreptomycin sulphate at a single dose of 20, 30, 40 or 50 mg/kg BW intramuscularly was able to cure goats without creating carriers. Streptomycin was able to cure CCPP affected goats on day 3 both in natural and experimental infections besides that treated goats completely developed immunity to Mccp (Rurangirwa et al. 1981a). Then CCPP inoculated goats were treated with oxytetracycline or tylosin by El Hassan et al. (1984) which reduced severity, but 20% remained infectious. They used short acting oxytetracycline and tylosin at a dose rate of 10 and 20 mg/kg BW intramuscularly, respectively daily for 6 days, and single dose of long acting oxytetracycline at 20 mg/kg BW intramuscularly. Afterwards several antibiotics were used in clinical cases and outbreaks. Danofloxacin has shown good effectiveness in clinical CCPP cases when administered at 6 mg/kg BW subcutaneously repeated at 48-h interval (Ozdemir et al. 2006). Long acting oxytetracycline has prevented disease and deaths and also controlled CCPP outbreak (Giadinis et al. 2008). Tiamulin was considered as one of the treatments of CCPP (Aytug et al. 1991) especially in early stages of the disease (AU-IBAR 2015; FAO 2017), but field availability limited its use. With passage of time antibiotics were evaluated for efficacy to Mccp for better utilization in CCPP and preventing the emerging threat of antibiotic resistance. The antibiotic sensitivity test (AST) based on minimum inhibitory concentration (MIC in μg/ml) in resistant mutants of mycoplasma species (*M. bovis*) included oxytetracycline (>32), enrofloxacin (2.5–32), tylosin (8–1024), and in some Mycoplasma mycoides cluster field isolates antibiotics tested were florfenicol (2.3–6), tylosin (1.3–6.6), ceftiofur (0.8–1.2), sulphamonomethoxine (2.3–7.3), erythromycin (1.7–6.7), lomefloxacin (3.3–6.7), and ofloxacin (6.7–9.3) (Wang et al. 2014; Chernova et al. 2016). However, some older but regularly used antibiotics like tetracycline or tylosin are believed to be effective when applied early in the infected herds (AU-IBAR 2015) where resistance is suspected. Currently, Mccp strains are susceptible to a wide range of antibiotic classes including macrolides, lincosamides, streptogramins, fluoroquinones and tetracyclines. However, some strains which are resistant to tetracyclines, erythromycin or streptomycin have been identified (Manso-Silván et al. 2011; EFSA AHAW Panel et al. 2017; Yatoo et al. 2018) indicating the possibility of antibiotic resistance in future if the appropriate measures are not taken. The better strategy will be alternating antibiotics in areas where there is no or least option of not using antibiotics (Yatoo et al. 2018). Even if antibiotics are to be used novel members or antibiotics need to be explored and combination therapy for therapeutic management need to be evaluated.

We have also evaluated antibiotics, namely tylosin, oxytetracycline and enrofloxacin at a dose rate of 10, 10 and 5 mg per kg BW, respectively through intramuscular route 48 hourly for three times in a period of 6 days in CCPP-affected Pashmina goats in Changthang, Ladakh, India in different trials. Tylosin gave better results than oxytetracycline followed by enrofloxacin in terms of recovery of animal, improvement in clinical parameters, and resolution of clinical and pathological signs. However, tylosin is rarely available to farmers in this region. Though oxytetracycline gave comparable results and has shown prophylactic effects against CCPP, it has resulted in deaths and deformity of kids in some observations when given to pregnant goats which might be due to extensive use over decades due to ease of availability and lack of knowledge regarding side effects. Besides, our preliminary study revealed less sensitivity to oxytetracycline on AST which might be due to long-term and uncontrolled usage by farmers than enrofloxacin. Enrofloxacin was quite effective and easily available but has not been applied under field conditions in the past in this trans-Himalayan mountainous region. Besides side effects of enrofloxacin including bone defects in neonates should be borne in mind for long term application especially in pregnant animals. Long acting preparations of antibiotics were preferred by nomadic herdsmen not only due to their long action, but also convenience in giving single injection which also minimizes labour and expenses.

Antibiotic-based treatment provides temporary kind of relief but for better therapeutic exploration and preventing threat of antibiotic resistance, minimum use of antibiotics should be practiced and where not feasible AST should be recommended before using. If the use of antibiotics is unavoidable, for example, remote livestock rearing areas of Asia and Africa, alternating effective antibiotics can be adopted. However, better is prevention by vaccination so as to avoid frequent and large-scale use of antibiotics and involvement of huge therapeutic costs.

## 7. Vaccination

Vaccination has been an important aspect of CCPP prevention and control in countries where it is prevalent since a large population of goats is affected and native populations are at an increased risk of outbreaks. However, in most of these countries, vaccines are not available and in some only laboratory-oriented research has been undertaken. Further, in order to prevent global spread of CCPP and to overcome ineffectiveness of antibiotics for treating CCPP, vaccines are imperative. Development of vaccines from local isolates, specific species, subspecies or strains and their potent antigens need to be explored for better and highly specific immune response. Many trials have also been conducted in this direction globally and few have been successful. Initially, MacOwan and Minette (1978) started vaccine trials for the disease using a high passage mycoplasma strain F38 and studied the effect on disease course. This was followed by a series of trials by Rurangirwa et al. (1981b, 1984, 1987a,b) who developed inactivated and saponin adjuvanated vaccines whose effectiveness have been proved under field conditions (Litamoi et al. 1989). However, the main drawback of these vaccines was their cost of production which was due to the fastidious nature of Mccp requiring rich and costly medium, slow growth to ensure a large quantity of medium for sustenance and requirement of heavy doses of antigen (0.15 mg per goat) for appropriate immune responses. King (1988) used formalinized culture as vaccine and reported that optimum age for vaccination of kids should be beyond 10 weeks of age. Rurangirwa et al. (1991) explored a lyophilised killed F38 vaccine for CCPP and found that this vaccine provided full protection against mortality and against clinical disease (95% protection). Different control strategies and vaccinations of CCPP have been detailed by Thiaucourt et al. (1996). As CCPP usually occurs in severe form and requires an early management of acute cases, some researchers have recommended the raising of antiserum against the pathogen (Wise et al. 1995; March et al. 2000).

Considering the limitations of whole culture vaccines, subunit fractions of Mccp as vaccines were studied for any inhibitory effects by March and Jones (1998) and March et al. (2000). Purification of IgG was also studied (Ey et al. 1978; March et al. 2000). Humoral immune responses were studied in experimentally Mccp infected goats by March et al. (2002). Aluminum hydroxide [Al(OH)_2_] terpene vaccine has also been recommended (Radostitis et al. 2009). Antibody response to Mccp vaccines was studied by Swai et al. (2013) and Lakew et al. (2014). In current times, novel vaccines based on strains or antigenic proteins are being evaluated. Ping et al. (2014) developed an inactivated vaccine (M1601 strain) for the disease. Thankappan et al. (2017) cloned and expressed P67 protein of *Mycoplasma leachii* which may have prophylactic value. Tesgera et al. (2017) evaluated an inactivated whole culture of the CCPP to be used as a trial vaccine. For evaluating vaccine quality control, Thiaucourt et al. (2018) used tandem mass spectrometry for identifying the mycoplasma antigen in reference to commercial CCPP vaccines.

Despite many research on CCPP vaccine development there are rarely vaccines available at commercial and field level especially in regions where the disease is a major problem in goat farming. Few CCPP vaccines are being manufactured in Africa (Ethiopia and Kenya), Middle East (Jordan, Saudi Arabia), Turkey and China. However, concerns have been raised regarding the quality of these vaccines when considering the European Pharmacopoeia guidelines (EFSA AHAW Panel et al. 2017; Thiaucourt et al. 2018). In India, a few trials have also been conducted on CCPP vaccines (Srivastava et al. 1990; Sikdar et al. 1993; IVRI 2017). Analysing the vast spread of CCPP among various countries, the development of more efficient, strain-specific and cheaper vaccines, especially the ones from local isolates, is imperative (EFSA AHAW Panel et al. 2017) under the prevailing situation of frequent CCPP outbreaks. This will also help in preventing the disease spread to other countries which are constantly under threat.

## 8. Conclusion and future perspectives

CCPP is a severe highly contagious respiratory mycoplasmal disease endangering goat farming in more than 40 countries of the world especially in Africa, Asia, Middle East and some European countries. The pathogen *Mycoplasma capricolum* subsp. *capripneumoniae* is highly fastidious and requires special and costly media for tedious culture and isolation, which need to be revised for convenience and cost-effectiveness. Clinical signs though related to pathogenesis in the lower respiratory tract, are often non-relevant and confused with other respiratory diseases. Pathogenic mechanisms are not clearly understood. However, glycolipid membrane and enzyme induced inflammatory and oxidative cascade of the respiratory tract epithelium and pleura need to be explored. Diagnosis need to be made farmer friendly, less cumbersome, cheap, and readily available with more convenience and rapidity by the application of novel diagnostic techniques enabling field applicability. Culture of Mccp, though a gold standard, is very difficult, lengthy and costly process and is not a very realistic approach that can have a widespread use. Among the serological tests, the GI test and CFT have been ranked the least sensitive for confirmation of clinical cases whereas indirect haemagglutination assay and cELISA are considered suitable, while the cELISA, LAT and PCR are the recommended tests. The LAT is rapid, simple, and better test for field and real time diagnosis applicable to whole blood or serum and is more sensitive than the CFT and easier than the cELISA. Refinement of sero-molecular diagnostic techniques, with special focus on convenience and field applicability, should be of utmost importance for future strategies.

For a better therapeutic regime, antibiotics need to be used judiciously and sensitivity of Mccp for them need to be evaluated periodically from conventional macrolides, or oxytetracyclines to novel antibiotics (fluoroquinolones) for effective treatment and minimizing risk of antibiotic resistance. Prevention and control are better achieved through prophylaxis. Vaccination needs advancement from conventional inactivated vaccines to subunit and recombinant vaccines exploring specific local isolates with most potent antigens (capsular glycolipids or proteins or cellular enzymes or proteins) for better immune response and requirement for single vaccination at the most not less than annually. Global efforts are required for combating this emerging and severe threat to goat farming globally over by devising appropriate diagnostic, prophylactic and therapeutic regimes for prevention and control of CCPP and OIE, FAO, AU-IBAR, EFSA AHAW and national or regional research stations have important role to play in future also.
